# Host-Pathogen Interactions after Lung Transplant: Are Cystic Fibrosis Patients Unique?

**DOI:** 10.1016/j.xcrm.2020.100061

**Published:** 2020-07-21

**Authors:** Eric D. Morrell, Erika D. Lease

**Affiliations:** 1 VA Puget Sound Heath Care System, Seattle, WA 98109, USA; 2 Harborview Medical Center, Seattle, WA 98109, USA; 3 University of Washington Medical Center, Division of Pulmonary, Critical Care, and Sleep Medicine, University of Washington, Seattle, WA 98195, USA

## Abstract

The impact of *Pseudomonas aeruginosa* airway colonization on lung allografts is not entirely clear. In this issue of *Cell Reports Medicine*, Dugger et al.^1^ identify distinct clinical outcomes and lung allograft biology in recipients with and without cystic fibrosis.

## Main Text

Lung transplantation is one option to potentially prolong or improve quality of life for many patients with cystic fibrosis (CF); however, long-term survival is limited by chronic lung allograft dysfunction (CLAD). CLAD is the most common cause of death in lung transplant recipients after the first year following transplantation and is found in 50% of recipients by 5 years and 75% by 10 years.^2^ *Pseudomonas aeruginosa* (PsA) colonization and infection are risk factors for the development of CLAD;^3^ however, the role of PsA on lung transplant outcomes in patients with CF is less clear.^4^, ^5^, ^6^, ^7^ Cystic fibrosis transmembrane conductance regulator (*CFTR*) gene mutations may lead to distinct immune responses in CF lung transplant recipients,^8^ especially when coupled with the unique airway microbiome that CF patients harbor. In this issue of *Cell Reports Medicine*, Dugger et al.^1^ present findings that reinforce the notion that patients with CF have distinct graft biology compared with other lung transplant recipients and could potentially respond differently to broadly applied clinical interventions.

The impact of PsA airway colonization on clinical outcomes after lung transplantation is not entirely clear, particularly in recipients with CF. In an early study, CF lung transplant recipients had more frequent PsA colonization and histologic evidence of infection versus non-CF recipients, although death from PsA infection was not different between the two groups.^9^ Pan-resistant PsA in CF lung transplant recipients has been associated with worse outcomes in some,^10^ but not all, reports.^4^^,^^5^^,^^7^ Confounding factors such as age and center-specific management protocols have made registry-based epidemiologic studies comparing CF versus non-CF lung transplant recipients difficult.

The report by Dugger et al.^1^ provides evidence that the host-PsA relationship may be distinct in CF versus non-CF lung transplant recipients. First, they present epidemiologic data from a single-center cohort of lung transplant recipients (n = 396) showing that CF status significantly modifies the relationship between PsA airway colonization and the development of CLAD or death (PsA colonization was associated with CLAD/death in non-CF patients but was not associated with CLAD/death in CF patients). Why might the relationship between PsA colonization and clinical outcomes in CF versus non-CF lung transplant recipients be different? RNA-sequencing data from epithelial cells isolated from airway brushings from these subjects demonstrates decreased expression of type I interferon and inflammatory gene sets in CF recipients colonized with PsA versus non-CF recipients colonized with PsA. Dugger et al.^1^ go on to show that primary airway epithelial cells (AECs) cultured in an air-liquid interface model from CF lung transplant recipients have decreased expression of interferon-related genes compared with non-CF recipients, and that increased neutrophil chemokine supernatant concentrations from native CF AECs are attenuated in post-transplant CF AEC supernatants.

What might be the mechanism for an attenuated epithelial immune response in grafts from CF lung transplant recipients versus non-CF recipients? Epigenetic analyses demonstrate increased DNA methylation around type I interferon promoters in AECs cultured from CF versus non-CF allografts. Finally, the authors show that a higher proportion of non-CF lung transplant recipients have non-mucoid PsA strains than CF patients, and that these strains are associated with increased risk of CLAD or death. It is possible that mucoid strains of PsA may trigger persistent allograft epigenetic changes in CF lung transplant recipients that lead to a dampened epithelial immune response. A key strength of this study is the integration of multiple layers of epidemiologic, translational, and *in vitro* data to build a coherent story that the host response to PsA infection is distinct in CF versus non-CF lung transplant recipients.

Despite these strengths, this study has several significant limitations and leaves some unanswered questions. First, the epidemiologic results reported are from a single-center cohort and will need to be validated in separate cohorts. Although the size of this cohort is relatively large for lung transplant studies, there were only 35 CF patients included. Moreover, 23% of the original cohort was not included because these subjects did not have a bronchoalveolar lavage performed during the study period (and thus did not have PsA culture data available). It is unclear how exclusion of these subjects may have influenced the results. Second, it is possible that a distinction between colonization versus infection may have contributed to the decrease in type I AEC expression in the CF PsA group versus the non-CF PsA group. Histologic and quantitative culture data is not available; however, all AECs from the non-CF PsA group were isolated from patients treated with antibiotics versus only 44% in the CF PsA group. It will be important to clarify whether colonization with PsA, independent of infection, elicits distinct AEC responses in CF versus non-CF recipients. Finally, more evidence is emerging that defective CFTR function may impact non-epithelial cells such as circulating immune cells.^8^ The possible contribution of non-epithelial cells to the development of CLAD in CF versus non-CF lung transplant recipients should be examined in future studies.

What are the broader implications of the findings reported by Dugger et al.^1^ to the lung transplant community? Precision medicine has revolutionized fields such as oncology, and a move toward precision medicine in the field of lung transplantation will require us to embrace complex microbiologic and host factors such as those described by Dugger and associates. Do non-CF lung transplant recipients merit different immunosuppression strategies compared with CF recipients? Should patients colonized with mucoid versus non-mucoid PsA strains be monitored differently? Does having CF influence these post-transplant monitoring strategies? The first step to identifying subtypes of lung transplant recipients that have distinct responses to treatment is uncovering differences in biology between different subgroups of lung transplant patients. The study by Dugger et al.^1^ is an important step in this direction.
